# HAPPINESS LOVES COMPANY? OLDER ADULTS’ POSITIVE AFFECT ACROSS LAYERS OF THE MOMENTARY SOCIAL CONTEXT

**DOI:** 10.1093/geroni/igae098.0140

**Published:** 2024-12-31

**Authors:** Alyssa Goldman, Sara Moorman

**Affiliations:** Boston College, Chestnut Hill, Massachusetts, United States; Boston College, Chestnut Hill, Massachusetts, United States

## Abstract

Positive affect supports physical and mental health as well as longevity. Ecological momentary assessments (EMAs) have been used to demonstrate that positive affect is significantly shaped by the quality and quantity of momentary social interactions that take place during everyday activities. We consider that positive affect may also be a function of the broader momentary interpersonal social environment, including the presence of acquaintances and unfamiliar others in everyday social spaces – social actors whose presence is typically not explored in studies of the social context. Using 15,379 EMAs from 378 older adults in the Chicago Health and Activity Space in Real-Time study in mixed effects regression models, we find that older adults experience more positive affect when they are with a social companion versus being alone, consistent with prior research. More novel, however, we find that when older adults spend time in social spaces where other people are present, they report significantly higher levels of momentary positive affect, regardless of whether they are with a personal social companion or alone, and regardless of the activity in which they are engaged. The magnitude of the effect of being in more occupied social spaces is approximately half the size of the effect of being in the presence of a social companion. We discuss the implications of the results for ongoing efforts to address social isolation among the older adult population and consider how the relevance of social actors beyond interpersonal ties could inform theory on the social climate of well-being and aging.

